# Klotho retards renal fibrosis through targeting mitochondrial dysfunction and cellular senescence in renal tubular cells

**DOI:** 10.14814/phy2.14696

**Published:** 2021-01-19

**Authors:** Jinhua Miao, Jiewu Huang, Congwei Luo, Huiyun Ye, Xian Ling, Qinyu Wu, Weiwei Shen, Lili Zhou

**Affiliations:** ^1^ State Key Laboratory of Organ Failure Research National Clinical Research Center of Kidney Disease Division of Nephrology Nanfang Hospital Southern Medical University Guangzhou China; ^2^ Bioland Laboratory (Guangzhou Regenerative Medicine and Health Guangdong Laboratory) Guangzhou China

**Keywords:** Klotho, mitochondrial dysfunction, renal fibrosis, senescence, Wnt/β‐catenin

## Abstract

Chronic kidney disease (CKD) has a high prevalence worldwide and is an intricate issue to whole medical society. Renal fibrosis is the common pathological feature for various kinds of CKD. As an anti‐aging protein, Klotho is predominantly expressed in renal tubular epithelial cells. Reports show Klotho could retard age‐related renal fibrosis. Mitochondrial dysfunction plays an important role in cellular senescence. However, the role of Klotho in mitochondrial dysfunction in CKD has not yet been determined. In this study, we treated unilateral ischemia‐reperfusion (UIRI) mice and cultured human renal tubular epithelial cells (HKC‐8) with Klotho. We assessed renal fibrosis, cellular senescence, and Wnt/β‐catenin signaling. We also focused on mitochondrial function assessment. In UIRI mice, ectopic expression of Klotho greatly retarded fibrotic lesions and the activation of Wnt/β‐catenin signaling. Interestingly, Klotho significantly preserved mitochondrial mass, inhibited mitochondrial reactive oxygen species (ROS) production and restored the expression of mitochondrial respiration chain complex subunits. Consequently, Klotho restrained cellular senescence. In HKC‐8 cells, Klotho significantly inhibited Wnt1‐ and Wnt9a‐induced mitochondrial injury, cellular senescence, and fibrotic lesions. These results suggest Klotho has a protective role in renal function through targeted protection on mitochondria. This further broads the understanding of the beneficial efficacies of Klotho in CKD.

## Graphical abstract

Our studies suggest Klotho has a protective role in renal function through targeted protection on mitochondria. This further broads the understanding of the beneficial efficacies of Klotho in CKD.

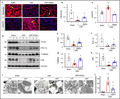

## 1 INTRODUCTION

The morbidity of CKD is in highly increasing rate and compels nephrologists to face the great difficulties for blankness of effective therapeutic strategies (Isakova et al., 2019; Legrand et al., 2019). Although a series of drugs such as angiotensin receptor blocker and calcium channel blocker (Woo et al., 2013) or the new drug FG‐4592, an oral hypoxia‐inducible factor prolyl hydroxylase inhibitor (Provenzano et al., 2016), show the therapeutic potential in the progression of CKD, however, there is still a high incidence of end‐stage renal disease (ESRD) in the coming 10 years (McCullough et al., 2019). The reason lies in the multifactorial mechanisms of CKD are not determined in detail. Recently, reports show that the loss of Klotho in plasma and kidney serves as an important inducer in the progression of CKD (Kim et al., 2013) and the associated cardiac disease (Silva et al., 2019), mineral bone disorder (Yamada & Giachelli, 2017), and metabolic disorder (Kim et al., 2019).

Klotho is mainly expressed in normal kidneys and was identified as an anti‐aging protein two decades ago (Kuro‐o et al., 1997). Recently, Klotho has been found to possess the potent efficacies on lowering inflammation (Zhou et al., 2015) and oxidative stress (Wang et al., 2012), activating autophagy (Shi et al., 2016), and preserving the stemness of progenitor cells (Sahu et al., 2018). Notably, Klotho serves as an endogenous inhibitor against Wnt/β‐catenin signaling (Zhou et al., 2013), the notorious criminal in driving podocyte injury (Zhou & Liu, 2015) and sustaining fibroblast activation (Xiao et al., 2016) through the induction of multiple signaling pathways such as renin–angiotensin system (RAS) (Zhou & Liu, 2016b) and the endopeptidase matrix metalloproteinase‐7 (MMP‐7) (He et al., 2012). Both of the latters are major players in promoting and accelerating kidney diseases and their fatal complications (Xie et al., 2016; Yang et al., 2019).

As a developmental signal, Wnt/β‐catenin signaling becomes inactive after birth; however, it is reactivated in various kinds of CKD (Luo et al., 2018; Miao et al., 2019; Xiao et al., 2016). Although Wnt/β‐catenin activation triggers the fibrotic injury in renal tubular epithelial cells (Zhou et al., 2013), the authentic cellular mechanisms involved are not fully elucidated. Recently, we found that Wnt/β‐catenin activation could trigger cellular senescence in tubular epithelial cells (Luo et al., 2018). Furthermore, Wnt/β‐catenin‐induced mitochondrial dysfunction such as the loss of mitochondrial mass, and increase in mitochondrial fragmentation and ROS production, plays the very important role in tubular cell aging and age‐related renal fibrosis (Miao et al., 2019). Although Klotho preserves mitochondrial function and mitigates tubular cell senescence in aged kidneys, its roles in these processes under CKD condition are not reported.

CKD presents many features such as mitochondrial damage and senescence in tubular epithelial cells (Bai et al., 2019; Luo et al., 2018), and flare‐up of inflammation as well as matrix deposition, to be a clinical manifestation of premature aging (Stenvinkel & Larsson, 2013). As a major type of kidney parenchymal cells, tubular epithelial cell plays a vital role in water and electrolyte reabsorption and secretion (Wang & Kestenbaum, 2018). The high abundance of mitochondria in renal tubular cells not only ensures the normal life activities in tubular cells but also affects the whole kidney function, since senescent tubular cells could produce large amounts of senescence‐associated secretory phenotype (SASP) components, such as proinflammatory and matrix‐synthesizing cytokines, to arouse renal fibrosis (Acosta et al., 2013; Luo et al., 2018). Thus, to find a modulator targeting against mitochondrial dysfunction and cellular senescence in renal tubular cells is of great significance to prevent and control the progression CKD.

In this study, we assessed the protective effects of Klotho in CKD mice and cultured renal tubular epithelial cells. The supplementation of Klotho greatly restored the imbalanced mitochondrial dynamic and definitely inhibited cellular senescence in tubular cells. Our results suggest Klotho has a protective role in renal function through targeted protection on mitochondria. This further broads the understanding of the beneficial efficacies of Klotho in CKD.

## 2 MATERIALS AND METHODS

### 2.1 Animal model

**FIGURE 1:**
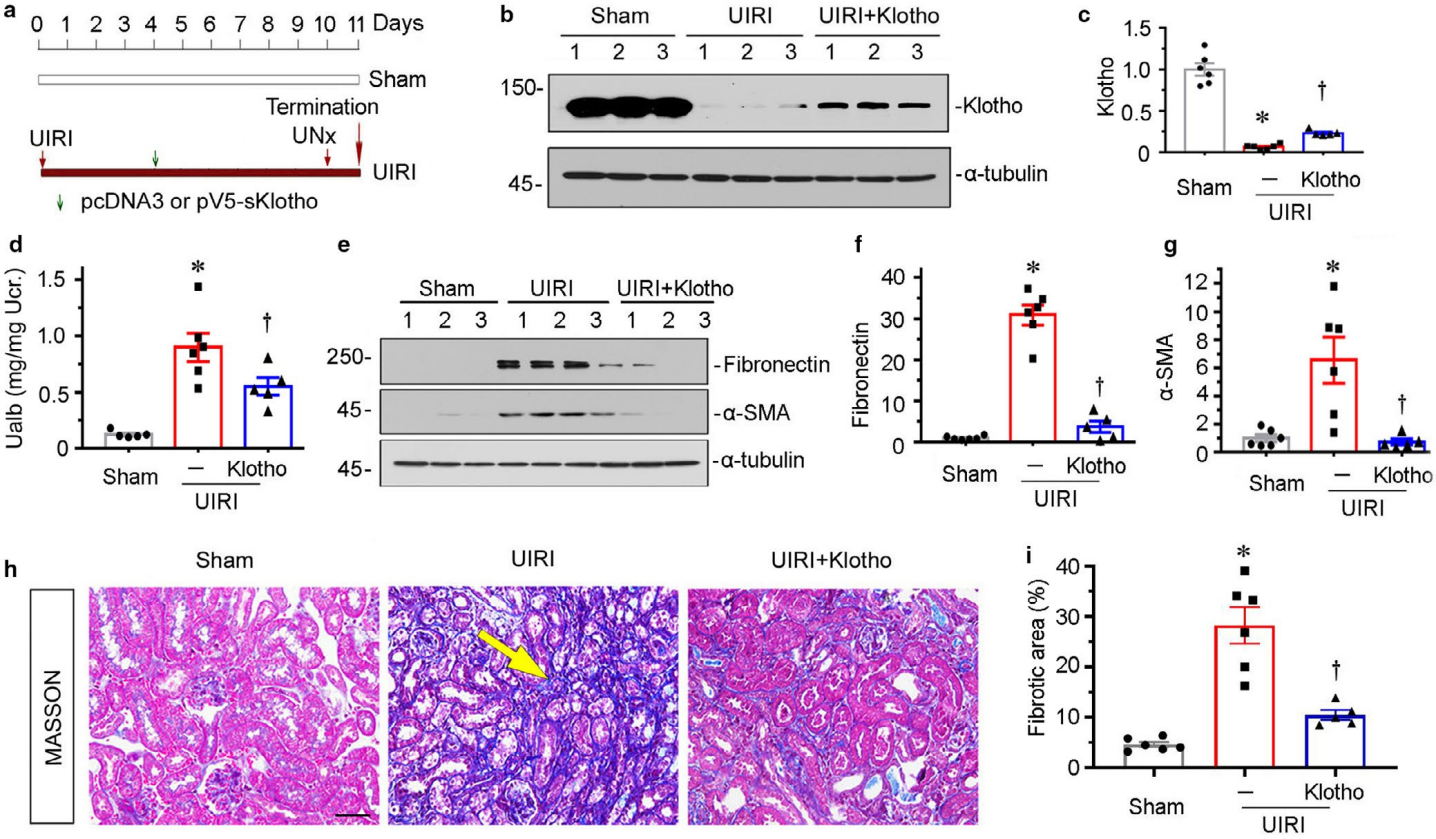
Ectopic expression of Klotho preserves kidney function and inhibits renal fibrosis in unilateral ischemia‐reperfusion (UIRI) mice. (a) Experimental design. Green arrow indicates the injection of empty vector (pcDNA3) or V5‐tagged, secreted Klotho expression vector (pV5‐sKlotho). Red arrows indicate the timing of renal IRI or nephrectomy surgery. (b) Representative western blots show renal expression of Klotho in three groups, as indicated. (c) Graphical representation of Klotho protein expression levels in three groups. *p < 0.05 versus sham controls; ^†^ *p* < 0.05 versus UIRI alone (n = 5–6). (d) Ualb levels in three groups, as indicated. Ualb was expressed as milligrams per milligram urinary creatinine. *p < 0.05 versus sham controls; ^†^ *p* < 0.05 versus UIRI alone (n = 5–6). (e) Representative western blots show renal expression of fibronectin and a‐SMA in three groups, as indicated. (f and g) Graphical representations of (f) fibronectin and (g) a‐SMA expression levels in different groups. *p < 0.05 versus sham controls; ^†^ *p* < 0.05 versus UIRI alone (n = 5–6). (h) Representative micrographs show collagen deposition in different groups, as indicated. Paraffin sections were subjected to Masson Trichrome staining. Arrow indicates positive staining. Scale bar, 50 μm. (i) Graphical representation of kidney fibrotic lesion in different groups after quantitative determination. *p < 0.05 versus sham controls; ^†^ *p* < 0.05 versus UIRI alone (n = 5–6). Ucr, urinary creatinine; UNx, unilateral nephrectomy.

A unilateral ischemia‐reperfusion (UIRI) surgery was performed in C57BL/6 (male, 8‐week‐old) mice (Southern Medical University Animal Center, Guangzhou, China), as previously reported (Zhou et al., 2018). First, the ischemia injury was performed on left renal pedicle using a microaneurysm clamp and maintained at 37°C by a temperature‐controlled heating system. Thirty‐five minutes later, the clamp was removed, and the reperfusion of left kidney was visually confirmed. Ten days later, the right kidney was to resect integrally. Sham operation was carried out only by a surgical incision in abdomen and stitch up. The overexpression of Klotho in mice was performed through a rapid (<5 s) injection of plasmid solution (2 ml) into the tail vein, a gene delivery approach as previously reported (Zhou et al., 2013). The animal experimental design is shown in Figure 1a. The secreted Klotho expression plasmid (pV5‐sKlotho) or empty vector (pcDNA3) was injected at 1 mg/kg by. All mice were sacrificed at 11 days after ischemia‐reperfusion injury. Serum, urine, and kidney samples were harvested for different analyses. All animal experiments were approved by the Animal Ethics Committee at the Nanfang Hospital, Southern Medical University.

### 2.2 Determination of urinary albumin level

The levels of urinary albumin (Ualb) in different groups were tested using a mouse Albumin ELISA Quantitation kit (Bethyl Laboratories, Inc., Montgomery, TX, USA). Urinary creatinine was assessed by an automatic chemistry analyzer (AU480; Beckman Coulter Inc, Kraemer Boulevard Brea, CA, USA). Ualb was normalized to creatinine.

### 2.3 Cell culture and treatment

Human proximal tubular epithelial cells (HKC‐8) were given by Dr. L. Racusen (Johns Hopkins University, Baltimore, MD, USA). Cell culture was performed as previously described (Zhou et al., 2018). The empty vector (pcDNA3), or Wnt1 expression plasmid (pHA‐Wnt1), or Wnt9a expression plasmid (pFlag‐Wnt9a) was transfected using Lipofectamine 2000 reagent (Invitrogen, Grand Island, NY, USA). Some cells were also treated with recombinant human Klotho (5334‐KL; R&D Systems).

### 2.4 Nuclear fraction isolation

Nuclear fractions were isolated using a commercial kit (BestBio, Shanghai, China), and performed according to the protocol provided by the manufacturer.

### 2.5 Western blot analysis

Western blot analyses were performed as previously reported (Liu et al., 2020; Zhou et al., 2019). Briefly, the kidney tissue or HKC‐8 cells were extracted the protein in lysis buffer. After the assessment of protein concentrations using a Bradford assay, proteins were subjected to SDS‐PAGE electrophoresis. The proteins were transferred into a PVDF membrane after electrophoresis, blocked in 1% bovine serum albumin for 1 hour, and then incubated with primary antibodies for 24 hours at 4°C. Furthermore, a secondary horseradish peroxidase‐conjugated antibody was added for 1 hour at room temperature. The antigen–antibody complexes were visualized using an ECL kit (Applygen, Beijing, China). The primary antibodies were as follows: anti‐Klotho (AF1819; R&D Systems), anti‐α‐SMA (A2547; Sigma‐Aldrich), anti‐fibronectin (F3648; Sigma‐Aldrich), anti‐GAPDH (RM2001; Ray Antibody Biotech, Beijing, China), anti‐Wnt1 (ab15251; Abcam), anti‐Wnt9a (ab176973; Abcam), anti‐β‐catenin (#610154; BD Transduction Laboratories, San Jose, CA, USA), anti‐PAI‐1 (AF3828; R&D Systems), PGC‐1α (ab54481; Abcam), anti‐Cytb (SAB1304939; Sigma‐Aldrich), anti‐TFAM (GTX112760; Genetex), anti‐p16^INK4A^ (ab189034; Abcam), anti‐p19^ARF^ (ab202225; Abcam), anti‐γH2AX (ab26350; Abcam), anti‐TOMM20 (ab186735; Abcam), anti‐p14^ARF^ (ab185620; Abcam), anti‐MMP‐7 (GTX104658; GeneTex Inc.), anti‐p53 (sc‐126; Santa Cruz Biotechnology), anti‐LRP6 (phosphor S1490) (ab76417; Abcam), anti‐α‐tubulin (RM2007; Ray Antibody Biotech, Beijing, China), anti‐Parp‐1 (9542S; Cell Signaling Technology), anti‐FasL (SC‐19681; Santa Cruz Biotechnology), anti‐β‐actin (RM2001; Beijing Ray Antibody Biotech), and anti‐TBP (ab818; Abcam).

### 2.6 Immunoprecipitation

The interaction of Klotho or LRP6 and Wnt9a in HKC‐8 cells was determined by co‐immunoprecipitation, as previously reported (Zhou et al., 2013). Cells were transfected with Wnt9a expression vector (pFlag‐Wnt9a) for 24 hours, and then co‐treated with recombinant human Klotho (5334‐KL; R&D Systems) for 12 hours. Cell lysates were immunoprecipitated overnight at 4°C with an antibody against Wnt9a (ab125957; Abcam) using protein A/G plus agarose (sc‐2003; Santa Cruz Biotechnology). After washing three times, the precipitated complexes were immunoblotted with anti‐Wnt9a (ab125957; Abcam), anti‐Klotho (AF1819; R&D Systems), and anti‐LRP6 (ab134146; Abcam) antibodies. Cell lysates were also tested by an antibody against GAPDH (RM2001, Ray Antibody Biotech).

### 2.7 Histology and immunohistochemical staining

Paraffin kidney sections (2 μm) were performed with Masson Trichrome staining (BA‐4079B; BASO). The immunohistochemical staining was performed on kidney sections (4 μm) (Zhou et al., 2018). After incubation by the primary antibodies, kidney sections were stained with the Vector M.O.M. immunodetection (Vector Laboratories, Burlingame, CA, USA). Antibodies used were as following: anti‐Wnt9a (ab189010; Abcam), anti‐Wnt1 (ab15251; Abcam), anti‐p16^INK4A^ (ab189034; Abcam), and anti‐caspase 3 (9662S; Cell Signaling Technology). The primary antibody was omitted as a negative control, and no staining occurred.

### 2.8 Immunofluorescence staining

Frozen kidney sections or cell coverslips were fist fixed in 4% paraformaldehyde, and then permeabilized by 0.5% of TritonX‐100/PBS, following the block by donkey serum incubation. The primary antibodies used were as following: anti‐fibronectin (F3648; Sigma, St. Louis, MO), anti‐active β‐catenin (19807 s; Cell Signaling Technology), anti‐γ‐H2AX (ab26350; Abcam), and anti‐TOMM20 (ab186735; Abcam).

### 2.9 SA‐β‐gal, mitoSOX, and MitoTracker staining

Frozen sections (3 μm) and HKC‐8 cell coverslips were assessed by the activities of senescence β‐galactosidase activity (#9860; Cell Signaling Technology), MitoTracker deep red (M22426; Thermo Fisher), or mitoSOX (M36008; Thermo Fisher) according to the manufacturer's instruction.

### 2.10 Transmission electron microscopy

The mitochondrial morphology of renal tubules and HKC‐8 cells were examined using ultrathin sections (60 nm). After fixation in 1.25% glutaraldehyde/0.1 mol/L phosphate buffer, the mitochondrial morphology was observed under an electron microscope (JEOL JEM‐1010, Tokyo, Japan).

### 2.11 Reverse transcriptase (RT) and real‐time PCR

Total RNA was extracted using a TRIzol RNA isolation kit (Life Technologies, Grand Island, NY, USA) and performed reverse transcription using AMV‐RT and random primers. Real‐time PCR was proceeded using a Platinum SYBR Green qPCR SuperMix‐UDG kit (Invitrogen). The sequences of the primers were as follows: mouse p19^ARF^, 5′‐CAATGTCCAAGATGCCTCCG‐3′ and 5′‐GCCCTCTCTTATCGCCAGAT‐3′; mouse γH2AX, 5′‐GGTGCTCGAGTACCTCACTG‐3′ and 5′‐CTTGTTGAGCTCCTCGTCGT‐3′; mouse NLRP3, 5′‐TTCTGAACCGAGACGTGAAGG‐3′ and 5′‐CTTGGCCTTGGCTTTCACTTC‐3′; and mouse β‐actin, 5′‐CAGCTGAGAGGGAAATCGTG‐3′, and 5′‐CGTTGCCAATAGTGATGACC‐3′.

### 2.12 Statistical analyses

All data were expressed as mean ± SEM. Statistical analysis was carried out using SPSS 25.0 (SPSS Inc, Chicago, IL, USA). Comparison between groups was made via one‐way analysis of variance followed by the Least Significant Difference or Dunnett's T3 procedure. *p* < 0.05 was considered as a significant.

## 3 RESULTS

### 3.1 Ectopic expression of Klotho preserves kidney function and inhibits renal fibrosis in unilateral ischemia‐reperfusion (UIRI) mice

To identify the effects of Klotho on renal fibrosis, we delivered the plasmid pV5‐sKlotho for secreted Klotho into UIRI mice through hydrodynamic‐based gene delivery, an approach that is commonly used to ectopically express proteins in liver and kidneys (Miao et al., 2019). The experimental design is shown in Figure 1a. Delivery of Klotho expression plasmid induced the restoration of Klotho expression in kidneys (Figure 1b,c). Furthermore, ectopic expression of Klotho significantly decreased the excretion of urine albumin (Figure 1d). Consistently, ectopic Klotho greatly blocked the upregulation of fibronectin and α‐SMA in UIRI mice (Figure 1, e through g). The similar results were observed when collagen deposition was tested by Masson Trichrome staining (Figure 1h,i).

### 3.2 Delivery of Klotho inhibits Wnt/β‐catenin signaling in UIRI mice

**FIGURE 2:**
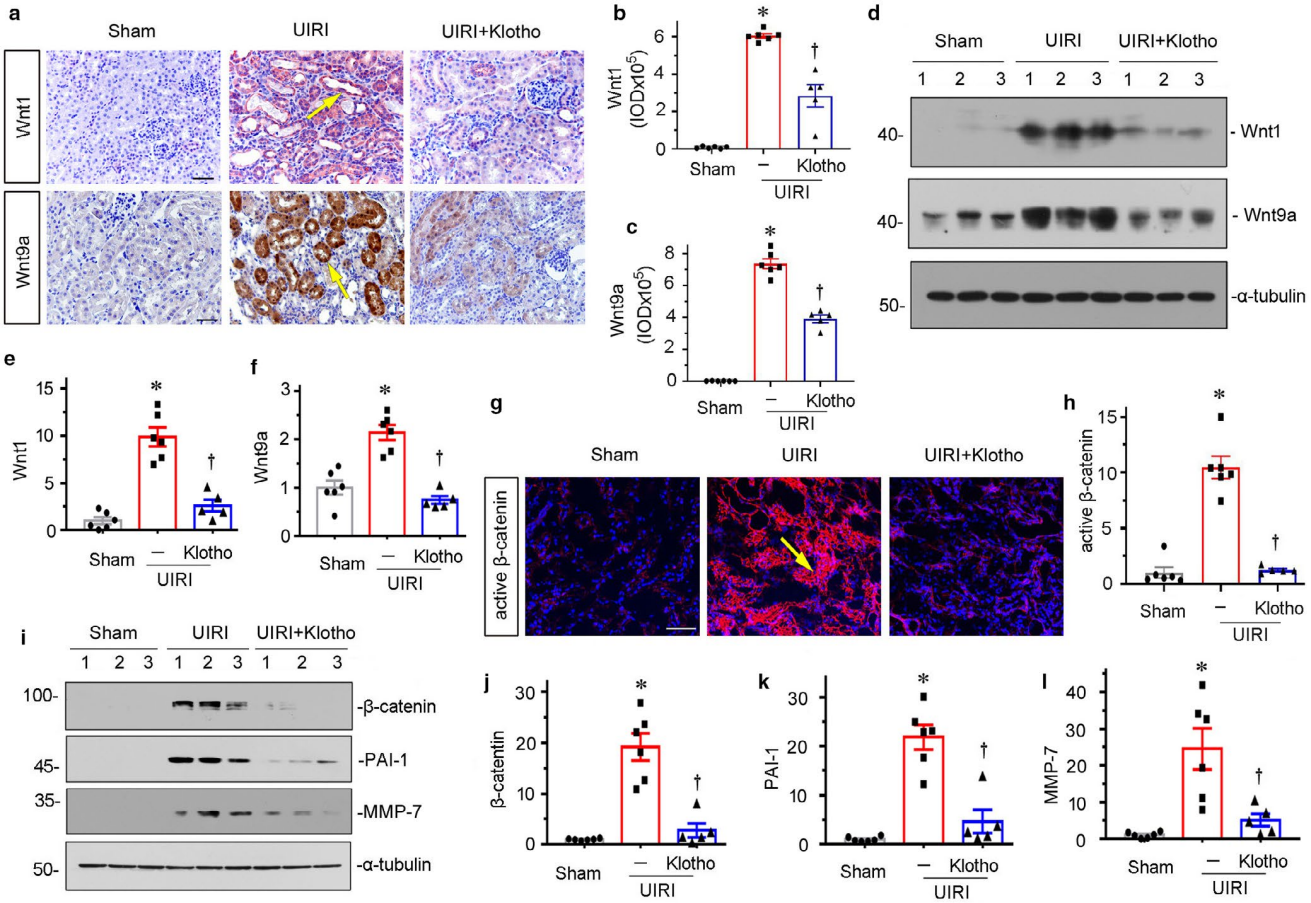
Delivery of Klotho inhibits Wnt/β‐catenin signaling in UIRI mice. (a–c) Representative micrographs and quantitative graphs show exogenous Klotho suppresses the expression Wnt1 and Wnt9a in UIRI mice. Arrows indicate positive staining. Scale bar, 50 μm. *p < 0.05 versus sham controls; ^†^ *p* < 0.05 versus UIRI alone (n = 5–6). (d) Representative western blots show Wnt1 and Wnt9a expression in three groups, as indicated. (e and f) Graphical representations of (e) Wnt1 and (f) Wnt9a protein expression levels in different groups, as indicated. *p < 0.05 versus sham controls; ^†^ *p* < 0.05 versus UIRI alone (n = 5–6). (g–h) Representative immunofluorescence micrographs and quantitative data show active β‐catenin expression in three groups. Arrow indicates positive staining. Scale bar, 50 μm. *p < 0.05 versus sham controls; ^†^ *p* < 0.05 versus UIRI alone (n = 5–6). (i–l) Representative western blot (i) and quantitative data show (j) β‐catenin, (k) PAI‐1, and (l) MMP‐7 protein expression levels in different groups, as indicated. *p < 0.05 versus sham controls; ^†^ *p* < 0.05 versus UIRI alone (n = 5–6).

We next assessed the role of Klotho in Wnt/β‐catenin signaling. The expression of Wnt1 was first examined by immunohistochemistry and western blotting analyses. As shown in Figure 2a,b, Wnt1 expression was greatly upregulated in UIRI‐affected kidneys, and mainly located in renal tubular epithelial cells. However, ectopic expression of Klotho largely inhibited the expression of Wnt1. The similar results were observed when Wnt1 was tested by western blotting analyses (Figure 2d,e). We then analyzed the expression of Wnt9a, an important player in renal tubular senescence (Luo et al., 2018). As shown in Figure 2a, c, d and f, Wnt9a was induced in UIRI mice, but significantly inhibited by ectopic expression of Klotho. As β‐catenin mediates the downstream pathway of Wnt signaling, we assessed the expression of β‐catenin and its targets PAI‐1 and MMP‐7. As shown in Figure 2g,h, the active form of β‐catenin was evidently upregulated in UIRI mice but largely blocked by ectopic expression of Klotho. The similar results were observed when β‐catenin and its targets PAI‐1 and MMP‐7 were assessed by western blotting analyses (Figure 2, i through l).

### 3.3 Klotho protects mitochondrial function in kidneys

**FIGURE 3:**
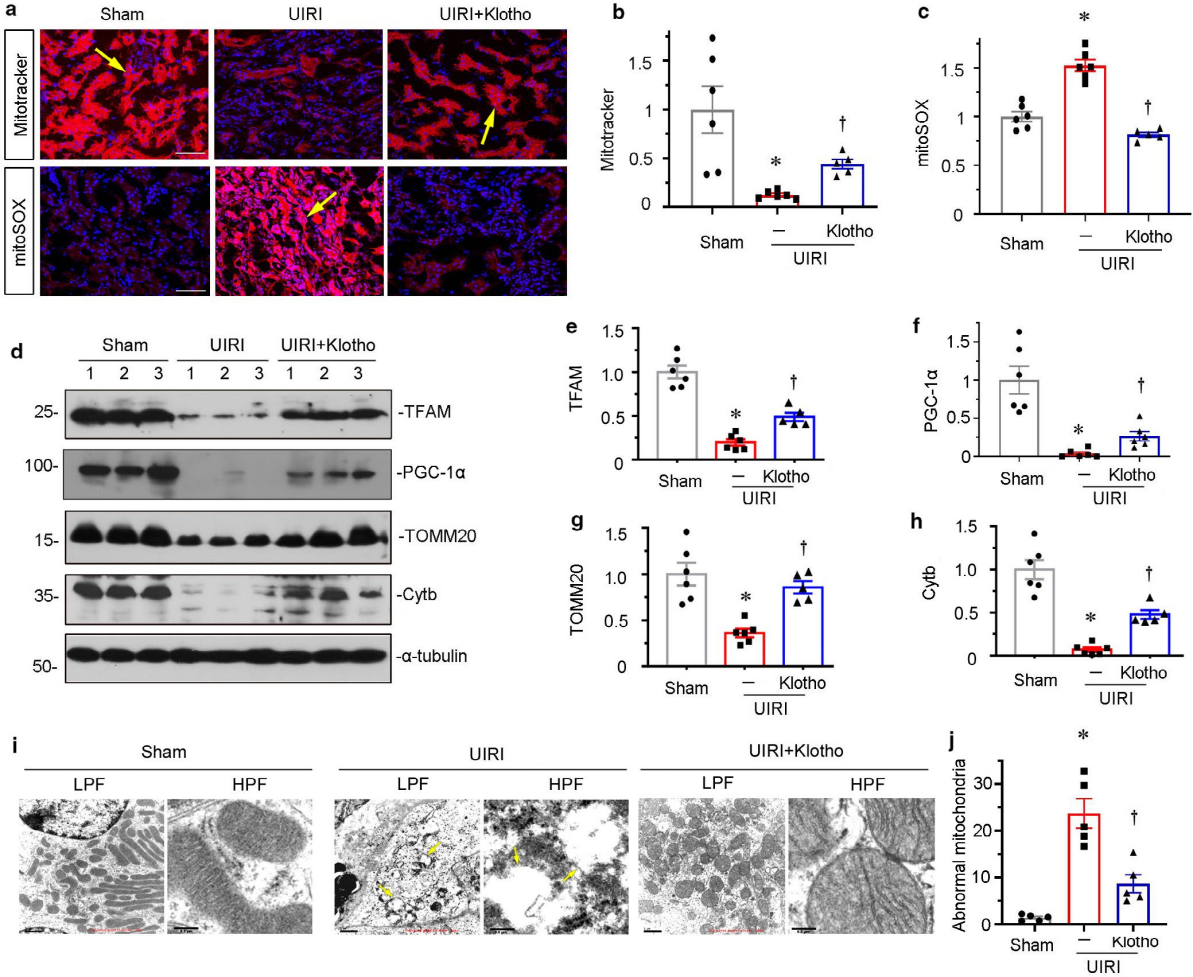
Klotho protects mitochondrial function in kidneys. (a–c) Representative micrographs and quantitative graph show mitochondrial mass and ROS generation in three groups. Mitochondria mass was assessed by Mitotracker staining. Mitochondrial ROS production was detected by MitoSOX staining. DAPI (4′,6‐diamidino‐2‐phenylindole) was used to stain the nuclei (blue). Arrows indicate positive staining. Scale bar, 50 μm. *p < 0.05 versus sham controls; ^†^ *p* < 0.05 versus UIRI alone (n = 5–6). Mitochondrial mass and mitochondrial ROS production levels were determined by the rate of MitoTracker deep red or MitoSox fluorescence intensity normalized to DAPI. (d–h) Representative western blot (d) and graphical representations of (e) TFAM, (f) PGC‐1α, (g) TOMM20, and (h) Cytb protein expression levels are shown. *p < 0.05 versus sham controls; ^†^ *p* < 0.05 versus UIRI alone (n = 5–6). (i and j) Representative micrographs and quantitative graph show mitochondrial ultrastructure morphology. Ectopic expression of Klotho protects against UIRI‐induced mitochondrial swelling, massive vacuolization, and cristae disorganization (arrows) in renal tubular cells. Images with lower (LPF) and higher (HPF) magnifications are shown. Scale bar in LPF, 1 μm. Scale bar in HPF, 200 nm. TEM, transmission electron microscopy. Five renal tubular epithelial cells per mouse were analyzed for the quantification of abnormal mitochondria in different groups. *p < 0.05 versus sham controls; ^†^ *p* < 0.05 versus UIRI alone (n = 5).

Since mitochondrial homeostasis plays an important role in sustaining normal kidney function, we assessed the role of Klotho in retarding mitochondrial dysfunction. Mitochondrial mass and mitochondrial ROS levels were assessed by Mitotracker deep red and mitoSOX staining, respectively. As shown in Figure 3, a through c, Klotho significantly reversed the loss of mitochondrial mass and decreased the production of mitochondrial ROS in UIRI mice. We then analyzed the expression of mitochondrial transcription factor A (TFAM) and peroxisome proliferator‐activated receptor‐γ coactivator‐1α (PGC‐1α), the two mitochondrial biogenesis‐related factors (Miao et al., 2019). As shown in Figure 3, d through f, ectopic expression of Klotho significantly restored the expression of TFAM and PGC‐1α. Similarly, the expression of the outer mitochondrial membrane protein, translocase of outer mitochondrial membrane complex subunit 20 (TOMM20), and OXPHOS complex III subunit cytochrome b (Cytb) were also preserved by ectopic expression of Klotho (Figure 3d, g and h). To further prove the protective role of Klotho in mitochondrial function, we next tested mitochondrial ultrastructure. As shown in Figure 3i,j, compared with the swollen mitochondria with disorganized and fragmented cristae in UIRI mice, Klotho could markedly ameliorate the abnormal structure of mitochondria. These results suggest Klotho presents an important role in protecting mitochondrial function in renal tubular cells.

### 3.4 Klotho inhibits tubular cell senescence in UIRI mice

**FIGURE 4:**
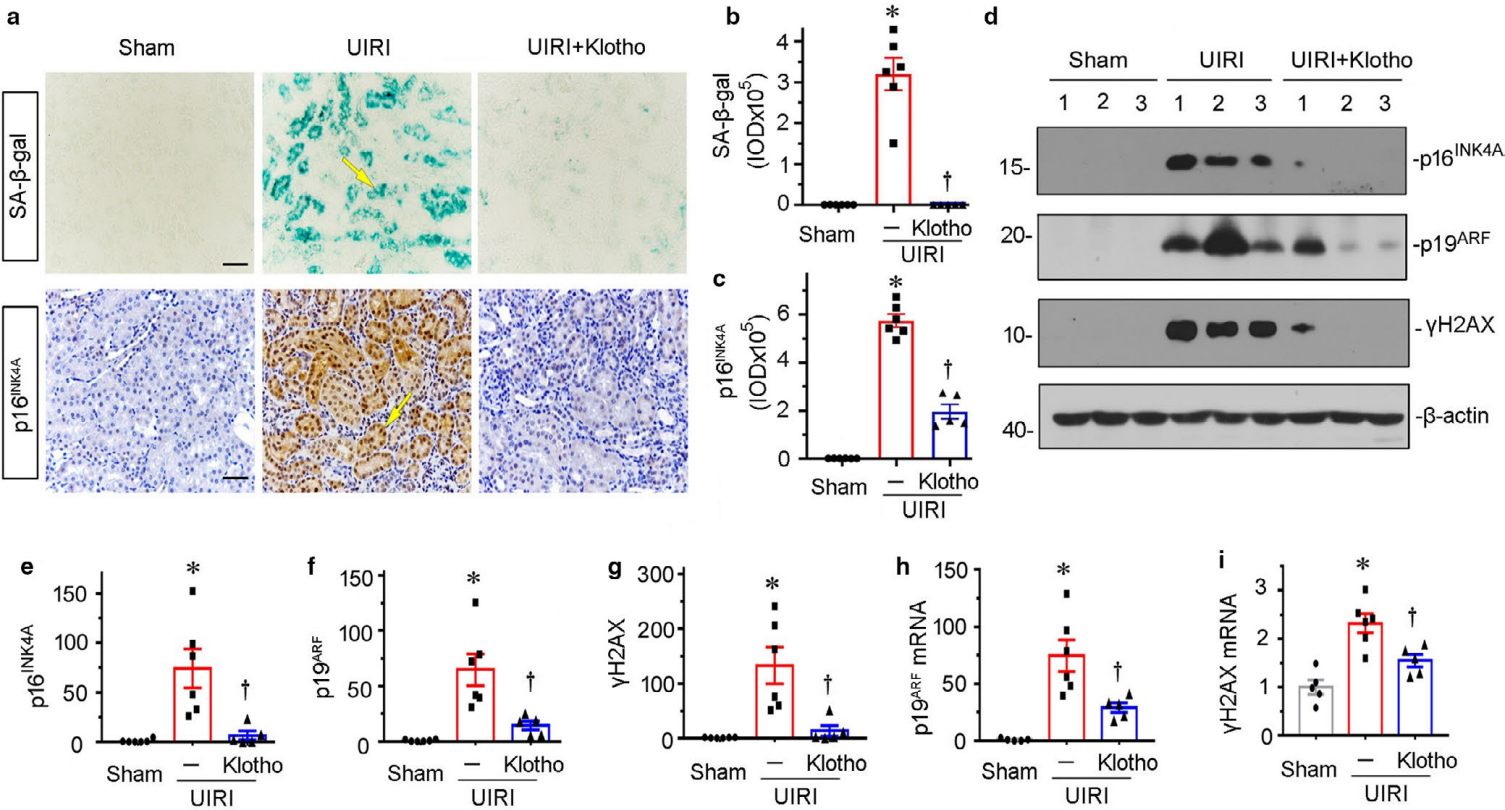
Klotho inhibits tubular cell senescence in UIRI mice. (a–c) Representative staining micrographs and quantitative graph show SA–β‐gal activity and p16^INK4a^ expression in different groups. Frozen kidney sections were stained for SA–β‐gal activity, which appears as bright‐blue granular staining in the cytoplasm of tubular epithelial cells. Paraffin sections were immunostained with an antibody against p16^INK4a^. Arrows indicate positive staining. Scale bar, 50 μm. *p < 0.05 versus sham controls; ^†^ *p* < 0.05 versus UIRI alone (n = 5–6). (d) Representative western blots show renal expression of p16^INK4a^, p19^ARF^, and γH2AX in three groups, as indicated. (e–g) Graphical representations show the expression levels of (e) p16^INK4a^, (f) p19^ARF^, and (g) γH2AX in three groups. *p < 0.05 versus sham controls; ^†^ *p* < 0.05 versus UIRI alone (n = 5–6). (h and i) Relative mRNA abundance of (h) p19^ARF^ and (i) γH_2_AX in different groups is shown. *p < 0.05 versus sham controls; ^†^ *p* < 0.05 versus UIRI alone (n = 5–6).

We next assessed tubular senescence by analyzing p16^INK4A^ expression and senescence‐associated β‐galactosidase (SA‐β‐gal) activity, the two important markers showing cellular senescence (Luo et al., 2018; Miao et al., 2019). As shown in Figure 4, a through c, UIRI surgery induced the increase in their expression in kidneys, especially in renal tubular epithelial cells. Ectopic expression of Klotho greatly decreased these effects. Furthermore, the expression of p16^INK4A^ and p19^ARF^, the other important senescence inducers (Luo et al., 2018), was assessed by western blotting analyses. As shown in Figure 4, d through f, p16^INK4A^ and p19^ARF^ were upregulated in UIRI mice but significantly inhibited by ectopic expression of Klotho. Consistently, the protein level of γH2AX, another important marker of DNA double‐strand breaks and cell senescence (Miao et al., 2019), was also significantly inhibited by ectopic expression of Klotho in UIRI mice (Figure 4d,g). The similar results were observed when p19^ARF^ and γH2AX mRNA levels were analyzed by real‐time PCR (Figure 4d, h and i). Interestingly, the tubular cell apoptosis was also inhibited by the administration of Klotho (Figure S1). As previously reported (Romero et al., 2016), senescent cells are generally resistant to apoptosis. Hence, we think the inhibitory effects of Klotho on tubular apoptosis are not related to its anti‐aging effects, but might be related to its other effects such as anti‐inflammatory effects (Zhou, Chen, et al., 2015). To test the hypothesis, we have checked the expression of NLR Family Pyrin Domain Containing 3 (NLRP3), an inflammasome marker protein (Mulay, 2019). The results show that Klotho could inhibit the mRNA expression of NLRP3 (Figure S1).

### 3.5 Klotho retards Wnt1‐induced cellular senescence through preserving mitochondrial function

**FIGURE 5:**
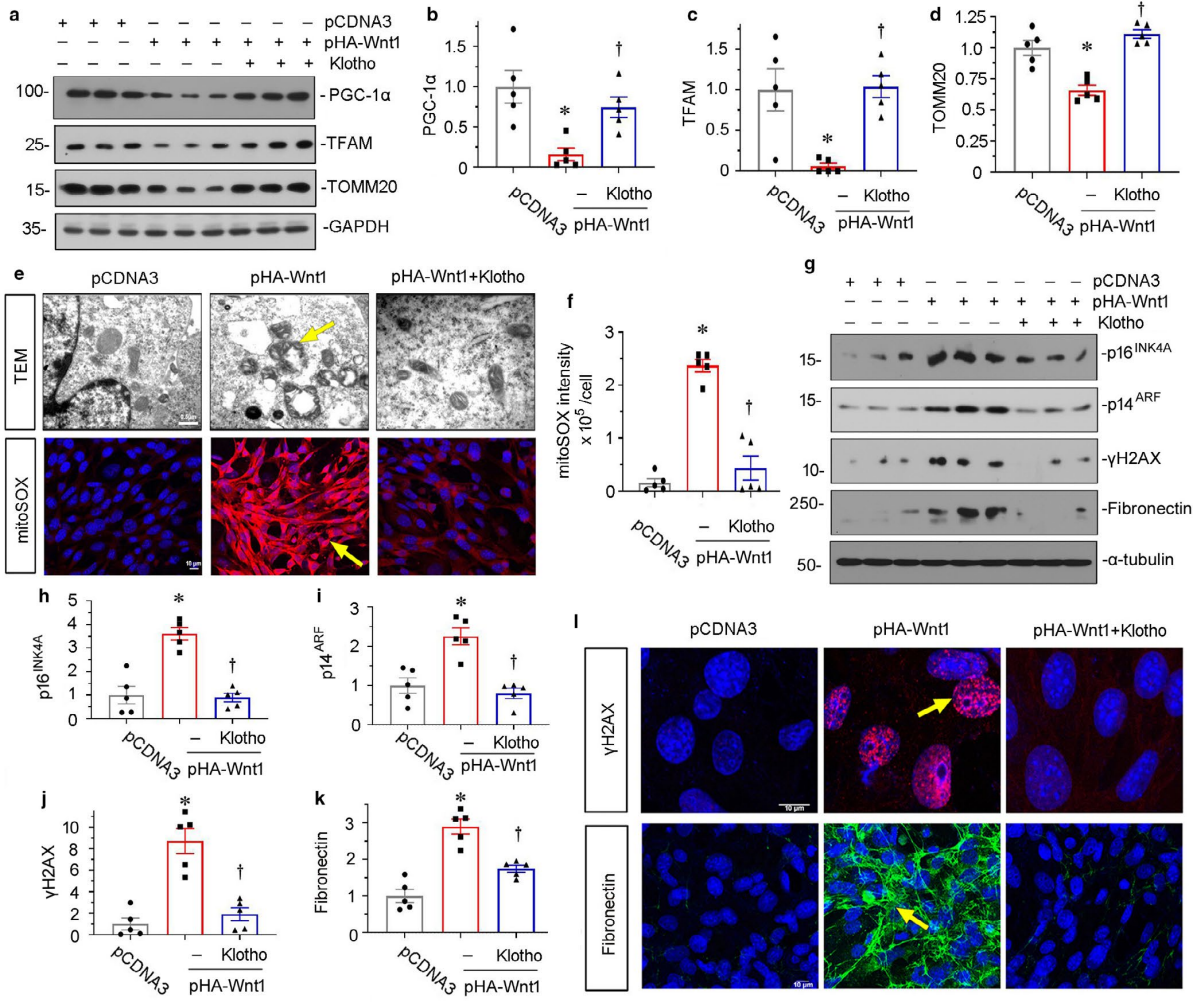
Klotho retards Wnt1‐induced cellular senescence through preserving mitochondrial function. (a) Representative western blots show the expression of PGC‐1α, TFAM, and TOMM20 in three groups, as indicated. HKC‐8 cells were transfected with empty vector (pcDNA3) or Wnt1 expression plasmid (pHA‐Wnt1) and then treated with recombinant human Klotho (100 ng/ml) for 24 hours. (b–d) Graphical representations of (b) PGC‐1α, (c) TFAM, and (d) TOMM20 protein expression levels in different groups, as indicated. *p < 0.05 versus pcDNA3; ^†^ *p* < 0.05 versus pHA‐Wnt1 alone (n = 5). (e) Representative micrographs show mitochondrial ultrastructure and ROS production. They were assessed by electron microscopy analyses and mitoSOX staining, respectively. Overexpression of Wnt1 induced mitochondrial vacuolization and cristae fragmentation and mitochondrial ROS production in renal tubular cells. Treatment with Klotho inhibited mitochondrial ROS production and prevented the disorder of mitochondrial structure. Arrows indicate positive staining. Scale bar in TEM, 0.5 μm. Scale bar in mitoSOX staining, 10 μm. (f) Quantitative graph show mitochondrial ROS generation in three groups. *p < 0.05 versus pcDNA3; ^†^ *p* < 0.05 versus pHA‐Wnt1 alone (n = 5). (g) Representative western blots show p16^INK4a^, p14^ARF^, γH2AX, and fibronectin expression in three groups, as indicated. (h–k) Graphical representations of (h) p16^INK4a^, (i) p14^ARF^, (j) γH2AX, and (k) fibronectin protein expression levels in different groups, as indicated. *p < 0.05 versus pcDNA3; ^†^ *p* < 0.05 versus pHA‐Wnt1 alone (n = 5). (l) Representative micrographs show the cells immunostained for γH_2_AX (red) and fibronectin (green), and counterstained with DAPI (blue). Arrows indicate positive staining. Scale bar, 10 μm.

We next assessed the effects of Klotho on mitochondrial function in cultured tubular cells. As shown in Figure 5, a through c, in HKC‐8 cells, overexpression of Wnt1 significantly inhibited the expression of PGC‐1α and TFAM, the two master regulators in mitochondrial biogenesis (Miao et al., 2019). However, co‐treatment with Klotho could significantly inhibit their downregulation. Consistently, Wnt1‐decreased TOMM20, an outer mitochondrial membrane protein, was also restored by co‐treatment with recombinant Klotho (Figure 5a,d). To further identify mitochondrial function, we assessed mitochondrial ultrastructure by TEM and mitochondrial ROS production by MitoSox staining. As shown in Figure 5e,f, overexpression of Wnt1 triggered the collapse of mitochondrial structure and production of mitochondrial ROS; however, these effects were ameliorated by co‐treatment with Klotho. Consistently, Wnt1‐induced upregulation of p16^INK4A^, p14^ARF^, and γH2AX, the three markers of cellular senescence (Luo et al., 2018), was significantly inhibited by co‐treatment with Klotho (Figure 5, g through j). Furthermore, Wnt1‐induced upregulation of fibronectin, a fibrotic injury marker, was also largely blocked by co‐treatment with Klotho (Figure 5g,k). The similar results were observed when γH2AX and fibronectin were tested by immunofluorescence (Figure 5l).

### 3.6 Klotho serves as an endogenous Wnt antagonist to block premature aging in tubular cells

**FIGURE 6:**
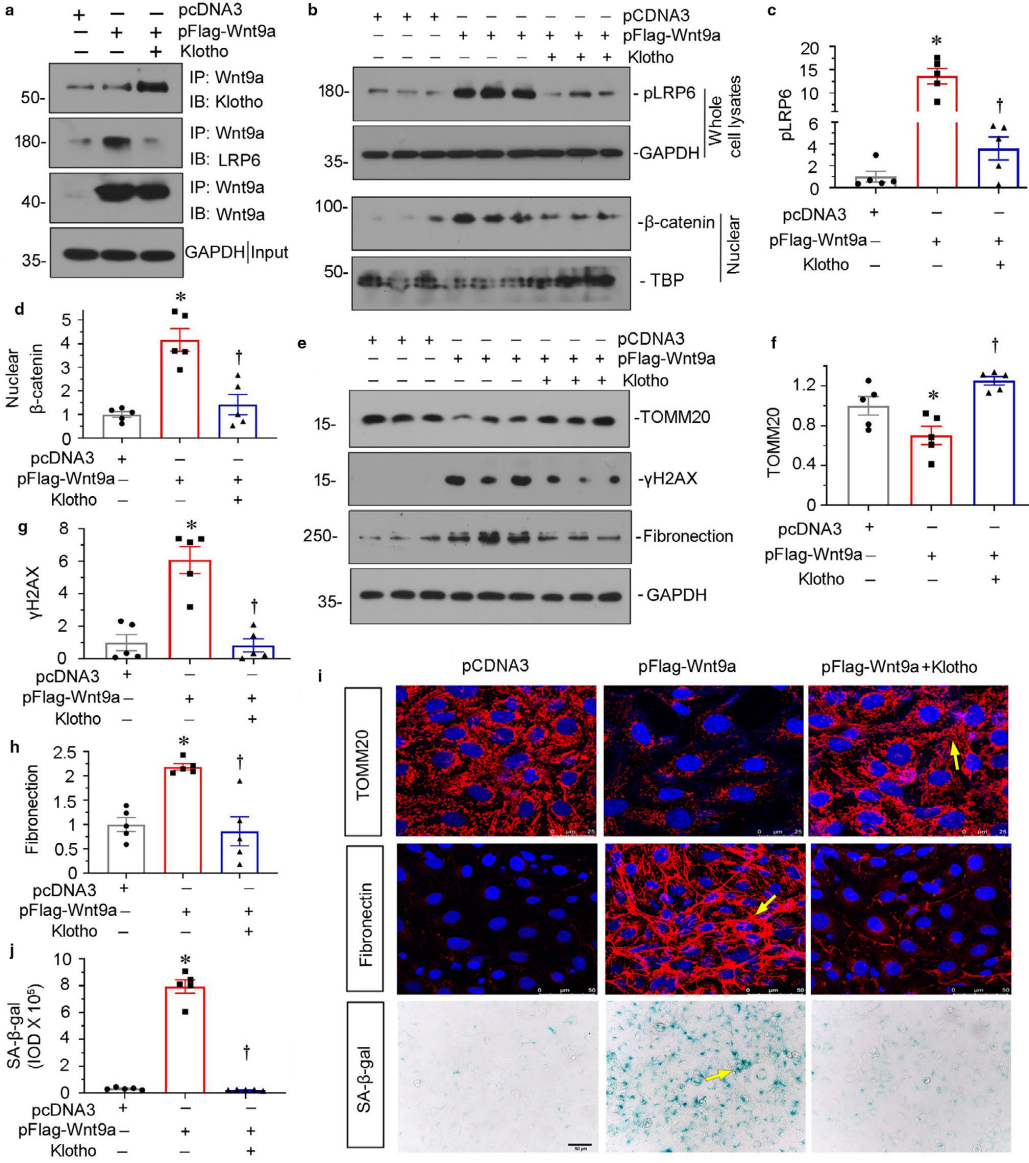
Klotho serves as an endogenous Wnt antagonist to block premature aging in tubular cells. (a) Co‐immunoprecipitation demonstrates that Klotho can bind to Wnt9a and competitively inhibits its combination with LRP6 in tubular epithelial cells. HKC‐8 cells were transfected with Flag‐tagged, Wnt9a expression vector (pFlag‐Wnt9a) and co‐treated with recombinant Klotho (100 ng/ml) for 12 hours. Cell lysates were immunoprecipitated with an antibody against Wnt9a, followed by immunoblotting with anti‐Klotho or anti‐LRP6 antibodies, respectively. (b) Representative western blots show the expression of pLRP6 and nuclear β‐catenin in three groups, as indicated. HKC‐8 cells were transfected with pcDNA3 or Wnt9a expression plasmid (pFlag‐Wnt9a) and then treated with recombinant human Klotho (100 ng/ml) for 12 hours. (c and d) Graphical representations show the expression of (c) pLRP6 and (d) nuclear β‐catenin in three groups. *p < 0.05 versus pcDNA3; ^†^ *p* < 0.05 versus pFlag‐Wnt9a alone (n = 5). (e–h) Representative western blots (e) and graphical representations show the expression of (f) TOMM20, (g) γH2AX, and (h) fibronectin in three groups. *p < 0.05 versus pcDNA3; ^†^ *p* < 0.05 versus pFlag‐Wnt9a alone (n = 5). (i) Representative micrographs show the expression of TOMM20, SA‐β‐gal activity, and fibronectin. Arrows indicate positive staining. (j) Quantitative graph show SA‐β‐gal activity in three groups. *p < 0.05 versus pcDNA3; ^†^ *p* < 0.05 versus pHA‐Wnt9a alone (n = 5).

Our previous report has proved Klotho could inhibit the activation of β‐catenin through binding to Wnt1, Wnt4, or Wnt7a (Zhou et al., 2013). However, the binding activity of Klotho with Wnt9a, a promoter in renal tubular epithelial cell senescence (Luo et al., 2018), is not determined. As shown in Figure 6a, Klotho could bind to Wnt9a, leading to decreased binding of Wnt9a with its co‐receptor LRP6. Furthermore, treatment with Klotho inhibited the phosphorylation of LRP6, an active form of LPR6 (Figure 6b,c). Consistently, Klotho retarded the nuclear translocation of β‐catenin (Figure 6b,d). As a result, treatment with Klotho significantly restored the expression of TOMM20, and inhibited the expression of γH2AX and fibronectin (Figure 6, e through h). Furthermore, the similar results were observed when TOMM20, SA‐β‐gal activity, and fibronectin were tested by staining (Figure 6i,j). These results further suggest Klotho could preserve mitochondrial function and inhibit cell senescence through blocking Wnt/β‐catenin signaling.

## 4 DISCUSSION

Klotho is an anti‐aging protein which mainly locates in adult kidneys (Zhou et al., 2013). Although Klotho could protect kidney function through inhibiting oxidative stress (Wang et al., 2012) and inflammation (Zhou, Chen, et al., 2015), the two important mediators in cellular senescence (Xiong & Zhou, 2019), the underlying mechanisms of Klotho in protecting kidneys are still not determined. Recently, Klotho was found to target inhibition on renin–angiotensin–aldosterone system (RAS) activity (Zhou, Mo, et al., 2015). Notably, RAS blockade could delay aging through protecting mitochondrial function (de Cavanagh et al., 2011). Our previous study also found RAS activation triggers the inability of mitochondrial biogenesis and induces mitochondrial damage, which further lead to age‐related renal fibrosis (Miao et al., 2019). These suggest the important role of RAS blockade in slowing aging. However, the effects of Klotho on mitochondrial protection and premature aging in CKD have not been reported.

CKD has a high morbidity and mortality in aged population (Minutolo et al., 2015), suggesting the intimate correlation between CKD and aging. Interestingly, CKD manifests a clinical feature of premature aging (Stenvinkel & Larsson, 2013). In an early onset stage of clinical nephropathy, the accumulated senescent tubular cells could be detected in patients with normal GFR (Verzola et al., 2008). Notably, tubular cell senescence is positively correlated with the fibrotic injury and decline in renal function in CKD (Luo et al., 2018). All of these suggest targeted inhibition of cellular senescence could be a very promising therapeutic strategy in prevention of CKD progression. Hence, to clarify the mechanisms of cellular senescence in renal parenchymal cells is especially important to open up a new route to treat CKD. Interestingly, our previous report has revealed mitochondrial dysfunction is intimately associated with the progression of aging in kidneys (Luo et al., 2018; Miao et al., 2019).

Mitochondrion serves as one of the key organelle to supply adenosine triphosphate (ATP), the essential substance for providing energy. As the major type of kidney parenchymal cells, renal tubular cells execute the reabsorption and excretion function of kidney. They possess extremely abundant contents of mitochondrion to sustain the high energy consumption. Mitochondrial damage not only results in energy absence but also triggers electron leaking to cause a great deal of ROS production to accelerate cell injury (Higgins & Coughlan, 2014). Recently, it was found that to preserve mitochondrial health stands in the center of prevention and control strategy of mitigating cellular senescence and aging (Theurey & Pizzo, 2018). Although RAS blockade shows the beneficial effects on mitochondrial protection (Sebastian et al., 2016), the current RAS inhibitors such as ACE inhibitors (ACEI) or AT1 receptor blockers (ARB) could not directly affect the synthesis of angiotensin II and stimulate renin secretion in a negative feedback mechanism (Zhou & Liu, 2016b). These would certainly restrain their slowing ability in aging. Hopefully, we have found that Wnt/β‐catenin signaling could masterly control the multiple genes of RAS (Zhou, Li, et al., 2015), suggesting the promising therapeutics of their inhibitors on mitochondrial dysfunction and cellular senescence. Interestingly, Klotho is an ideal endogenous antagonist to Wnt/β‐catenin signaling activation (Zhou et al., 2013). However, the real role of Klotho in CKD‐affected mitochondrial dysfunction and cellular senescence is not clarified.

In this study, we have found that supplement of Klotho could effectively ameliorate renal fibrosis (Figure 1) and the activation of Wnt/β‐catenin signaling (Figure 2). These effects were associated with the improvement of mitochondrial function. To assess mitochondrial function, we analyzed mitochondrial mass and the production of mitochondrial ROS. We also tested the expression of mitochondrial OXPHOS subunits and observed the ultrastructure of mitochondria by TEM. As shown in Figure 3, ectopic expression of Klotho could significantly preserve mitochondrial function and maintain the normal structure of mitochondria. Consequently, Klotho significantly protected against cellular senescence (Figure 4). To identify the underlying mechanism, we performed *in vitro* study. We proved that Klotho could significantly reverse mitochondrial damage and cellular senescence in Wnt1‐treated cells (Figure 5). As previous reported as Klotho binding to Wnt1 (Zhou et al., 2013), we also proved that Klotho could bind to Wnt9a, a mediator of tubular cell senescence (Luo et al., 2018), and inhibit the downstream signals of β‐catenin activation and premature aging in tubular cells (Figure 6). These results suggest Klotho could protect against tubular cell senescence through targeted inhibition on mitochondrial dysfunction in CKD. We could not exclude that other effects of Klotho such as anti‐inflammatory effects (Zhou, Chen, et al., 2015) could inhibit tubular cell apoptosis (Figure S1), which would synergistically ameliorate the progression of renal fibrosis. As tubular cell apoptosis is the cause primarily related to acute kidney injury (An et al., 2020), while cellular senescence and differentiation of renal tubular cells are more responsible for the development and progression of CKD (Luo et al., 2018; Yang et al., 2010; Zhou & Liu, 2016a). We think that the protection of Klotho on CKD and renal fibrosis would be more related to its inhibitory effects on cellular senescence.

Although it is previously reported that Klotho serves as an endogenous antagonist of Wnt/β‐catenin signaling (Kadoya et al., 2020; Zhou et al., 2013), protects mitochondrial function in aged cells (Miao et al., 2019; Sahu et al., 2018), and Klotho protects against renal fibrosis (Zhou, Mo, et al., 2015), the present study shows for the first time that Klotho strongly inhibit tubular cellular senescence and protects mitochondrial function in CKD mouse model. Our study further strengthen the mechanistic linkage of Wnt/β‐catenin signaling with mitochondrial function and aging (Miao et al., 2019). As an endogenous antagonist to Wnts, Klotho would play an important safeguarding role to some senescence‐promoting Wnts. Interestingly, we have also firstly proved that Klotho inhibited cell senescence through binding to Wnt9a, a senescence inducer in renal tubular cells (Luo et al., 2018).

Taken together, we hypothesize that the protective effect of Klotho on mitochondria attributes to its strong inhibitory effects on Wnt/β‐catenin signaling, as Wnt/β‐catenin signaling activation plays a crucial role in mediating mitochondrial dysfunction and age‐related renal fibrosis (Miao et al., 2019). Our results further identify the broad beneficial efficacies of Klotho in CKD.

## CONFLICTS OF INTEREST

The authors declared no competing interests.

## AUTHOR CONTRIBUTIONS

Jinhua Miao, Jiewu Huang, Congwei Luo, Huiyun Ye, Xian Ling, Qinyu Wu, and Weiwei Shen conducted the experiments and prepared the materials involved in this study. Lili Zhou conceived this study. Lili Zhou participated in its design and coordination. Lili Zhou, Jinhua Miao, Jiewu Huang, and Congwei Luo contributed to the analysis and interpretation of the data. Lili Zhou drafted the manuscript. All authors read and approved the final manuscript.

## Supporting information



Fig S1

## Data Availability

The data that support the findings of this study are available from the corresponding author upon reasonable request.
