# Supplementary figures and images for: Klotho retards renal fibrosis through targeting mitochondrial dysfunction and cellular senescence in renal tubular cells
Source: Physiol Rep. 2021 Jan 19;9(2):e14696. doi: 10.14814/phy2.14696 (PMC7814487; doi:10.14814/phy2.14696)

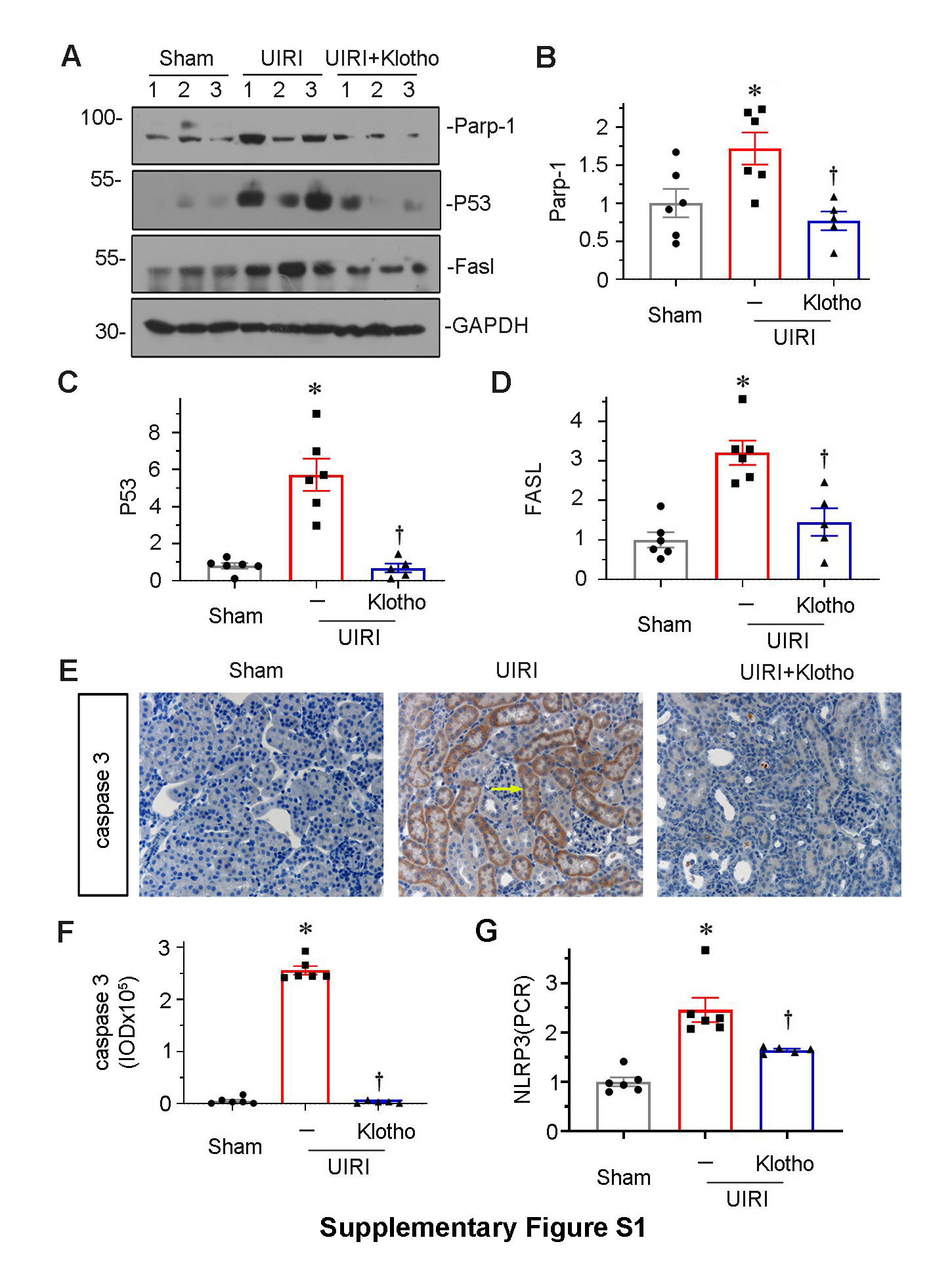

Supplement: Supplementary file 1 — Fig S1 [file PHY2-9-e14696-s001.jpg]
